# The Inclusion of Fathers in Parent Coaching Interventions for Young Autistic Children: A Systematic Review

**DOI:** 10.1007/s10803-025-06820-0

**Published:** 2025-04-10

**Authors:** Ciara Donohue, Fionnuala Tynan

**Affiliations:** 1 Department of Educational Psychology, Inclusive and Special Education, Mary Immaculate College, Trainee Educational & Child Psychologist, Board Certified Behaviour Analyst (BCBA), South Circular Road, Limerick, V94 VN26 Ireland; 2 Faculty of Education, Mary Immaculate College, Limerick, Ireland

**Keywords:** Autism, Early-intervention, Parent-coaching, Fathers

## Abstract

Parent-mediated interventions are fundamental in supporting parents of autistic children. In particular, parent coaching has emerged as a key component of such interventions. However, the characteristics of such interventions remain underexplored. Therefore, the current review aimed to synthesise characteristics of parent-coaching interventions for young autistic children (<6 years). Specifically, the review aimed to investigate the extent to which fathers are being included in parent-coaching interventions. A systematic review of the literature was conducted following PRISMA guidelines. A total of 5 studies which met the strict inclusion criteria were included in the final analysis. Studies were summarised regarding participant information, study design, intervention characteristics, dependent variables, study outcomes and social validity findings. Results revealed that only 2 fathers were represented in the included studies, which included 94 parent-child dyads overall. Although limited to two studies, outcomes indicated that parent-coaching can be successfully implemented with fathers. Findings concerning other intervention characteristics are also discussed. Overall, this review highlights the need for more in-depth research into fathers’ experiences of parent-coaching interventions and related child outcomes.

According to the American Psychiatric Association (APA, 2022), Autism Spectrum Disorder (ASD; hereafter referred to as autism) is a neurodevelopmental condition characterised by differences in social communication and the presence of specific and/or intense behaviours and/or interests. Other associated characteristics of autism include hypo- and hyper-sensitivities to various environmental stimuli, differences in executive functioning, and difficulties with sleep and emotional regulation (Blijd-Hoogewys et al., 2014; Peterson et al., 2015; Stepanova et al., 2017). The presence and intensity of such characteristics vary from one individual to another. Individuals identified as autistic each possess their own unique set of differences, strengths, and challenges that impact how they learn and interact with their environment (Bent et al., 2024).

Some early social communication differences observed in autistic children include social interest, joint attention, affect and imitation (Dow et al., 2017; Field, 2017; Fusaro et al., 2014; Sacrey et al., 2015). Typically developing children acquire such skills during the first two years of life through naturally occurring interactions, which are important in developing later skills such as vocal speech (DeQuinzio et al., 2016; Field, 2017; Hansen et al., 2018). With increasing prevalence rates of autism worldwide (Zeidan et al., 2022), early diagnosis has been described as crucial. The research suggests that support provided earlier in life leads to better outcomes regarding language, cognitive abilities, well-being and quality of life (Bent et al., 2024; Corsello, 2005; Jhuo & Chu, 2022; Webb et al., 2014). In addition, some research has shown that without effective early intervention to support skills such as functional and social communication, autistic children are more likely to engage in behaviours of concern to get their needs met (Koegel et al., 2014).

The support needs of autistic children vary significantly, and approaches to intervention also differ in terms of intensity, format and application (Bent et al., 2024). A balanced view of neurodiversity acknowledges the individual strengths of autistic children while also acknowledging the challenges they may experience, inherently or due to dynamic interaction with the environment (den Houting, 2019; Leadbitter et al., 2021). Interventions are typically strengths-based and focused on teaching useful skills and improving subjective quality of life (Chapman & Bovell, 2020; den Houting, 2019; Kapp et al., 2013). For example, intervention goals typically target life skills, identifying and supporting communication preferences and identifying triggers to behaviours of concern (Dawson et al., 2022; Kapp et al., 2013). Therefore, it is essential to identify evidence-based interventions that promote children’s early developmental skills and equip them with the skills they will need to navigate their world as they get older (Sandbank et al., 2020).

Naturalistic Developmental Behavioural Interventions (NDBIs; Schreibman et al., 2015) have emerged as a group of empirically validated interventions for young autistic children. Fundamental components of NDBIs include teaching in natural environments, child-initiated interactions, shared control and the use of naturally occurring opportunities and contingencies (Schreibman et al., 2015). Several intervention programs based on these components have been developed, including Pivotal Response Training (PRT; Koegel et al., 2016), the Early Start Denver Model (ESDM; Rogers & Dawson, 2020) and Joint Attention, Symbolic Play, Emotional Regulation (JASPER; Kasari et al., 2021). Such approaches have been shown to be effective in promoting the development of early social communication, language and play skills for autistic children (Harrop et al., 2017; Kasari et al., 2021; Mirenda et al., 2022; Randolph et al., 2011; van Noorden et al., 2022).

While clinicians can deliver numerous NDBIs, the involvement of parents in early intervention is strongly emphasised (Schuck et al., 2022). This involvement can facilitate the development of skills within the context of parent-child interactions and daily routines, thereby increasing exposure to the intervention and fostering parent empowerment (Pellecchia et al., 2020; Schreibman et al., 2015). Research indicates that one of the fundamental components of successful parent-mediated interventions is parent coaching (Pellecchia et al., 2020). Key elements of parent-coaching interventions include authentic learning opportunities, collaborative goal-setting, demonstration, in vivo feedback/guidance, and reflection (Pellecchia et al., 2020). Parent coaching in early intervention is considered an interactive process between a clinician and a parent or caregiver. It aims to actively equip parents with strategies to support their child’s development and participation in daily routines (Pellecchia et al., 2020; McDuffie et al., 2013). Effective parent coaching has enhanced parent responsiveness, self-efficacy, and treatment engagement (Estes et al., 2014; Kasari et al., 2014; McDuffie et al., 2013). Furthermore, a recent meta-analysis conducted by Heidlage et al. (2020) showed that parent-mediated interventions positively impacted children’s language and communication skills.

The growing emphasis on family-centered practice underscores the importance of supporting the entire family (Mas et al., 2022). However, mothers or female caregivers remain the prominent figures in early intervention (Flippin & Crais, 2011; McBride et al., 2017) and autism research more generally (Braunstein et al., 2013). For example, Braunstein and colleagues (2013) previously explored the inclusion of fathers in autism research. Findings revealed that the experiences of mothers outnumbered the experiences of fathers more than fivefold. Over the past number of years, there has been a shift in traditional family roles and fathers are spending more time caring for their children and are more directly involved than fathers of previous generations (Hodkinson & Brooks, 2023). However, this shift in caregiving practices has not been reflected in the research and fathers’ experiences remain scarce. The majority of parent-mediated interventions do not explore the role fathers play in effective intervention or the possible benefits of paternal involvement (Rankin et al., 2019). This was further highlighted in a review of parent-implemented interventions for autistic children by Flippin and Crais (2011). Findings showed that out of the 27 studies included in the review, only 3 reported including fathers as participants.

The literature on child development suggests that both mothers and fathers have unique and significant roles in their children’s development (Braunstein et al., 2013; Flippin & Crais, 2011; Pleck, 1997; Rankin et al., 2019; Sadownik, 2023). Historically, research has typically focused on mother-child interactions. The existing research indicates that father involvement is associated with numerous positive child and family outcomes, including higher academic achievement, increased educational and economic attainment, enhanced self-esteem and emotional well-being, and fewer problem behaviours among typically developing children (Deutsch et al., 2001; Flouri & Buchanan, 2004; Harris et al., 1998). Greater paternal involvement and stronger co-parenting among parents of autistic children may be particularly impactful due to the unique challenges of raising an autistic child (Kurzrok et al., 2021). Furthermore, an emerging body of research underscores the valuable role fathers can play in fostering their children’s early social communication skills (Pancsofar & Vernon-Feagans, 2006, 2010). Studies have shown that fathers exhibit distinctive play, interaction, and communication styles compared to mothers. For instance, fathers often adopt more direct communication styles and engage in more rough-and-tumble or physical play (Flippin, 2019; Flippin & Crais, 2011; Robinson et al., 2021). Such differences may be crucial for overall child development and, notably, for autistic children, who may display variations in their language, social, and play skills (Georgiou & Spanoudis, 2021). Therefore, enhancing the role of the father is essential in the context of play and communication-based interventions for autistic children for many reasons (Flippin & Crais, 2011).

Firstly, father participation may support positive child and parent outcomes. This was underscored in a study conducted by Louis and Kumar (2015). Fathers were shown how to engage in responsive and sophisticated interactions with their children. Results demonstrated that such interactions had positive outcomes on their children’s communication, play and social skills. The participating children were able to demonstrate improved communicative intent, respond constructively to social cues and participate in tasks of daily living. In addition, father involvement in parent-mediated interventions has implications in terms of overall family functioning. The involvement of both parents in their child’s intervention program has the potential to ameliorate parental stress and reduce the overall burden on mothers (Flippin & Crais, 2011; Kurzrok et al., 2021; Leiter, 2004). Studies that have included fathers have demonstrated increased father confidence in their parenting behaviour and self-esteem, while reductions in maternal stress levels have also been reported (Bendixen et al., 2011; Louis & Kumar, 2015).

Therefore, given the potential benefits of paternal involvement in parent-mediated interventions and the recommendation that fathers be included in such interventions, it is important to explore current practices in this area. It’s fundamental that clinicians and researchers develop a greater understanding of paternal involvement in interventions for autistic children, the extent to which they are involved and the outcomes of such interventions. Therefore, the current review sought to provide an up-to-date review of parent-coaching interventions for young autistic children and explore the extent to which fathers are being included in such interventions. In summary, the current review aims to answer the following research questions:

1. To what extent are fathers included in parent-coaching interventions?
2. What are the outcomes of parent-coaching interventions which include fathers in terms of intervention effectiveness and collateral effects on parental stress levels?

## Method

### Search Strategy

A systematic search of the literature was conducted using the following databases: Academic Search Complete, PsycINFO, PsycArticles, ERIC and Medline. As these databases frequently publish research on interventions for children with developmental differences, they were deemed the most appropriate. Search terms inputted into these databases are summarised in Table 1. Search terms were combined using the Boolean operations “AND” and “OR”.

**Table 1:** Summary of search terms for systematic literature review

Participants	Age Group	Setting	Interventionists	Intervention type
Autism OR Asperger OR ASD	Child* OR Toddler OR Baby* OR Babies	Home OR Early Intervention	Parent OR Mother OR Father OR Guardian	Coaching

### Data Selection Criterias

#### Inclusion Criteria

To be included in the current review, identified studies had to meet the following criteria: (i) all children had to have a diagnosis of Autism (by a qualified professional) and be aged 0–6 years; (ii) the intervention had to be implemented by the child’s parents or guardians; (iii) an experimental design had to be employed i.e. a single-subject research design, group research design or a randomised control trial (RCT); (iv) parents had to be coached to implement the target intervention using at least one parent-coaching strategy as defined by Pellecchia et al. (2020). These included; authentic learning opportunities, collaborative goal-setting, demonstration, in vivo feedback/guidance and reflection; (v) the intervention had to take place in the child’s home environment; (vi) the study had to report on the effect of the intervention on parent fidelity/use of taught strategies and child outcomes; (vii) the study had to be published in a peer-reviewed journal; (viii) all studies had to be published in English.

### Exclusion Criteria

Studies were excluded from the current review based on the following criteria: (i) children in the study were aged over 6 years 11 months; (ii) all children in the study did not have an official diagnosis of Autism. This included children who were described as “at risk of Autism”; (iii) the intervention was implemented by the researcher, a clinician or other professional; (iv) the study did not employ experimental conditions or was qualitative or correlational. Reviews and meta-analyses were also excluded; (v) the intervention did not include at least one coaching strategy as defined by Pellecchia et al. (2020); (vi) the intervention was carried out in a clinic, community setting or via telehealth. This included interventions delivered via apps; (vii) the study did not include measures of parent fidelity or child outcomes; (viii) the study did not focus on social communication skills e.g., focused on behaviour reduction/support; (ix) studies were not published in a peer-reviewed journal and; (x) the study was not published in English.

### Data Extraction

The articles were imported into referencing software (Mendeley v.2.122.1), which removed all duplicates. The first author screened all of the articles in the following phases: (i) title and abstract screening; (ii) full-text screening against the inclusion and exclusion criteria; and (iii) analysing all included studies for methodological quality.

### Methodological Design

The PRISMA 2020 (Page et al., 2021) guidelines for systematic reviews were followed throughout the screening process. The main data was extracted and summarised in Table 2 in line with PRISMA (Page et al., 2021) reporting guidelines. Findings from each study were summarised under the following headings: participants (sample size, diagnosis, age); caregiver involved; study design; intervention characteristics (type, length); dependent variables and study outcomes.

**Table 2:** Table summarising the main information extracted from the included studies

Reference	Participants •Sample size • Diagnosis •Age (months) • Gender	Care-giver Involved	Study Design	Intervention • Type • Length	Dependent Variables (DVs)	Study Outcome
Akemoglu and Tomeny (2021)	*n* = 3 ASD 38; 71; 44 3 M	Mother (3)	Multiple-baseline design across behaviours (MBD).	Parent Training (Two 1-hour sessions) Coaching (3–4 sessions for each strategy)	1. Fidelity of implementation of reading techniques and naturalistic communication teaching (NCT) strategies (modelling, mand-model & time delay) after training and coaching. 2. Children’s communicative behaviour– verbal & non-verbal.	Improvements in mothers use of reading techniques & naturalistic teaching strategies with high fidelity. The children initiated more communicative attempts when mothers used the time-delay. Results maintained at post- intervention follow-up.
Harrop et al. (2017)	Caregiver-mediated JASPER Group *n* = 43 ASD M = 30.7 (SD = 3.5) 35 M, 8 F Psych-education intervention (PEI) Group. *n* = 43 ASD M = 32.3 (SD = 2.7) 35 M, 8 F	Mother (86)	Randomised comparative design with 2 different conditions. 1. Joint Attention, Symbolic Play, Engagement and Regulation (JASPER): caregiver-mediated social-communication intervention. 2. PEI group	1. JAPSER Active coaching of the caregiver 1 h a week for 10 weeks. 2. PEI Education & support to parents via 1:1 meetings 1 h per week for 10 weeks.	1. Caregiver Child Play interaction (CCX). 2. Child Repetitive Restrictive Behaviours (RRB’s). 3. Care-giver responses to RRB’s. 4. Response success.	Child RRB’s remained stable in both groups from entry to exit. At 6 months follow both groups showed a significant increase in RRB’s. Caregivers from the JASPER group responded to a greater number of their child’s RRB’s and their responses were more successful.
Erturk et al. (2021)	*n* = 2 ASD 50; 52 2 M	Mother (2)	Multiple baseline design (MBD) across 3 behaviours.	Parent training (on target social communication skills) followed by parent coaching. (Coaching sessions continued until parents achieved 80% fidelity for 3 consecutive sessions– approx. 3 sessions per skill lasting between 15–45 min).	1. Parent treatment fidelity. 2. Behavioural requests for preferred items or activities. 3. Imitation of play actions. 4. Response to joint attention bids from parents.	Increased parent fidelity for one parent in two strategies and in all strategies for the other parent. All children showed improvements in target behaviours. Parents generalised skills to different toy sets and treatment effects maintained at follow up for both parents and children.
Flippin (2019)	*n* = 1 ASD 37 1 M	Father (1)	Multiple baseline across behaviours (MBD).	Twelve weekly coaching sessions lasting 1 h.	1. Proportion of father implementation of target responsive verbal & play strategies (follow-in comments, follow-in directives, responsive physical play). 2. Frequency of child expressive language production. 3. Pre-post ratings of parental stress.	The father quickly learned to use 3/4 targeted responsive strategies. Child use of single word utterances increased compared to baseline and some 2-word utterances were observed. Effects were maintained for 3/4 targeted responsive strategies. 1 and 2 word utterances were higher in maintenance than in baseline. Parenting stress decreased post-intervention.
Zaghlawan and Ostrosky (2016)	*n* = 2 ASD 37; 60 2 M	Father (1) Mother (1)	Multiple baseline across strategies design (MBD).	Modified reciprocal imitation training (RIT) using Parent Coaching (15 sessions)	1. Parents use of contingent imitation, descriptive language, object imitation & gestural imitation. 2. Children’s object & gestural imitation.	Both parents learned to use the intervention strategies via coaching. Both children showed improvements in their imitation skills.

### Quality Appraisal

The included studies were assessed for methodological rigour using Gough’s Weight of Evidence (WoE, Gough, 2007) framework. Each study was critiqued in three areas related to the quality of the study. This included the methodological quality (WoE A), the methodological relevance (WoE B) and the relevance of the evidence to the target research question (WoE C). The results of WoE A, B and C were then combined to establish an overall weighted score (WoE D). This determined the extent to which each study contributed evidence in addressing the target research questions.

WoE A was calculated using an appropriate quality appraisal tool. In the current review, studies adopted a mixture of single-subject and randomised comparative designs. Therefore, matching a quality appraisal tool reflective of each research design was essential to ascertain its methodological quality. Studies which employed a single-subject design were analysed using the Single-Case Experimental Design (SCED) Scale (Tate et al., 2008). The SCED scale is an 11-item rating scale for single-subject designs, with 10 items assessing methodological quality and use of statistical analysis. Studies which employed a randomised comparative design or randomised control trial (RCT) were analysed using the Critical Appraisal Skills Programme (CASP; 2021) checklist for randomised control trials. The presence or absence of the methodological criteria highlighted in these tools were assessed for each study and converted into WoE A quality rating and descriptive scores. Percentage WoE A quality scores were calculated by adding the total number of “Yes” answers, dividing it by the total number of items in the scale and multiplying this by 100.

The Gray (1996) matrix, as summarised by Petticrew and Roberts (2006), was used as a guideline to determine the methodological relevance of each study (WoE B). A summary of the scoring criteria and rationale used to determine the methodological relevance of each study (WoE B) can be found in Table 3.

**Table 3:** WoE B scoring criteria and rationale

WoE B Rating Score	WoE B Descriptive Rating	Research Design	Rationale
1	Low	Single-case research design studies	Do not allow for the effectiveness of an intervention or its components to be measured effectively (Gray, 1996; Petticrew & Roberts, 2006).
2	Acceptable	Quasi-experimental & Cohort Studies	Classified as appropriate research methodologies for evaluating the effectiveness of variables (Gray, 1996).
3	High	Randomised Controlled Trials	RCT’s are ‘gold standard’ when examining research questions related to ‘effectiveness’ (Petticrew & Roberts, 2006).

Weight of evidence C (WoE C) appraises the relevance of the findings in answering the review question. Gough (2007) emphasises the importance of the sample in answering the research question. As the present review aimed to investigate if fathers are included in parent-mediated interventions and related outcomes, this was seen as an essential criterion for answering the research question. A rubric including the coding protocol was developed to score the WoE C for each study and is presented in Table 4.

**Table 4:** WoE C scoring criteria

WoE C Score	Criteria
3 (High)	Participants in the study are fathers of autistic children. The study reports on the outcomes of father involvement in the parent-mediated intervention. This sample is highly relevant to the research question, thus findings are considered highly relevant to the review question.
2 (Medium)	Participants in the study include fathers of autistic children and other caregivers. The study reports on the outcomes of father or other caregiver involvement in the parent-mediated intervention. Findings are considered less relevant to the research question as the sample is mixed and not specifically targeted at fathers. Therefore findings are given less relevance.
1 (Low)	Participants in the study don’t include fathers of autistic children. This sample is less relevant to the review and findings are therefore considered less relevant.

Rating and descriptive scores from WoE A, B and C were combined to calculate the WoE D score. This determined the extent to which each study answered the research questions. Studies were classified as either “low”, “medium” or “high”. Studies classified as high were given more weight in answering the research questions, while studies classified as low were given less weight.

## Results

### Search Results

**Fig. 1:**
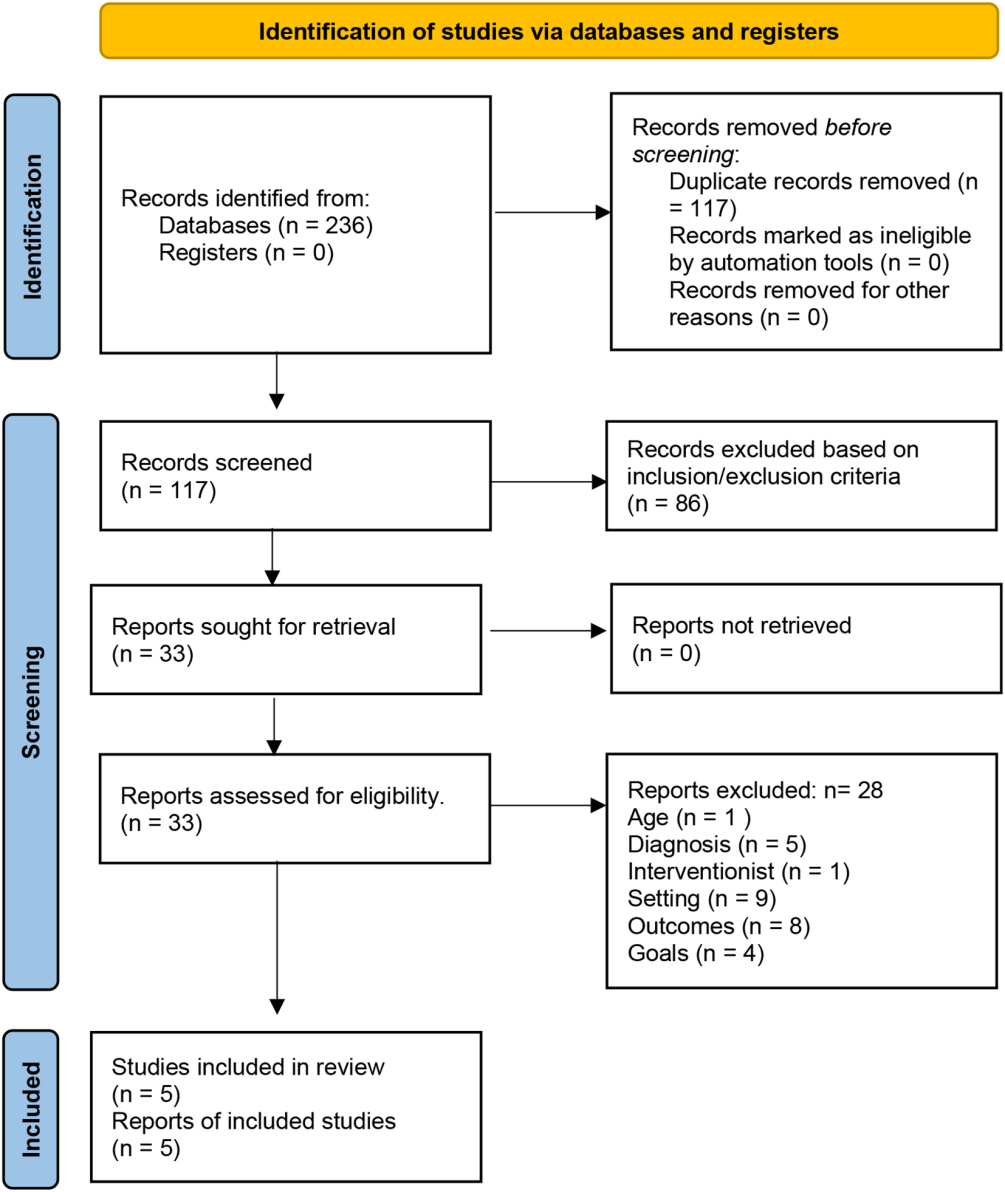
PRISMA flow diagram

The initial search yielded 236 articles; once duplicates were removed, 117 remained. Title and abstract screening was then completed, which led to the exclusion of 86 articles which did not meet the inclusion criteria. The remaining 33 articles were then full-text screened against the inclusion and exclusion criteria and were removed for the following reasons: nine studies were not carried out in the home environment; eight studies did not report on parent fidelity and/or child outcomes; 5 studies were excluded based on the child’s diagnosis. Many studies included children “at risk” of autism as participants. Other studies pooled data together from children with differing diagnoses e.g. Down Syndrome; four studies did not target social communication goals; one study was excluded based on the age of the included children and one study was excluded as the parents were not the interventionists. The PRISMA flow chart (Page et al., 2021) in Fig. 1 summarises the studies included and excluded at each stage of the screening process. Once full-text screening was complete, a total of 5 studies remained.

### Quality of the Included Studies

A summary of the Weight of Evidence (WoE) scores assigned at each stage of the quality appraisal stages is provided in Table 5. The studies which employed a single-subject design or an RCT were rated as “high” in terms of methodological quality (WoE A). In terms of WoE B, which assessed the methodological relevance of each study, four of the studies were rated as “low” and one study was rated as “high”. In terms of WoE C, which assessed the relevance of each study in answering the research questions, three studies were rated as “low”, 1 study was rated as “medium”, and 2 studies were rated as “high”. The results of WoE A, B and C were then combined to establish an overall weighted score (WoE D) for each study. This determined the extent to which each study contributed evidence in addressing the target research questions. All studies received an overall WoE D rating of “medium”.

**Table 5:** Summary of woe scores assigned to each study

Study	WoE A Score	WoE B Score	WoE C Score	WoE D Score
Akemoglu and Tomeny (2021)	3 (high)	1 (low)	1 (low)	2 (medium)
Erturk et al. (2021)	3 (high)	1 (low)	1 (low)	2 (medium)
Flippin (2019)	3 (high)	1 (low)	3 (high)	2 (medium)
Zaghlawan and Ostrosky (2016)	3 (high)	1 (low)	2 (medium)	2 (medium)
Harrop et al. (2017)	3 (high)	3 (high)	1 (low)	2 (medium)

### Participant Information

The number of parent-child dyads varied in each study. Some studies included one parent-child dyad (Flippin, 2019), while larger interventions included up to 86 parent-child dyads (Harrop et al., 2017). A total of 94 parent-child dyads were represented across the 5 studies. Out of these 94 dyads, 2 were father-child dyads, and the remainder were mother-child dyads (Flippin, 2019; Zaghlawan & Ostrosky, 2016).

### Study Design

The studies included in this review employed a variation of two different research designs: single-subject designs and randomised control trials (RCTs). One study employed a randomised comparative design (Harrop et al., 2017), which allowed for the effectiveness of a parent psychoeducation intervention and a parent-coaching intervention to be compared. The remaining studies employed a single-subject design, all of which were multiple-baseline designs (Akemoglu & Tomeny, 2021; Erturk et al., 2021; Flippin, 2019; Zaghlawan & Ostrosky, 2016).

### Intervention Characteristics

This review specifically looked at coaching as a method to support parents in implementing strategies to foster their child’s development. Different coaching strategies were used across the studies: providing information about the target skill, modelling, demonstration of the skills, feedback and reflection. The length of the intervention varied between studies or was not reported. This ranged from 3 to 4 sessions to 12 weeks. Erturk et al. (2021) used parental level of fidelity as a criterion for moving on, which affected the overall length of the intervention. Sessions typically lasted one hour, with some studies reporting 10–12 weekly 1-hour sessions (Flippin, 2019; Harrop et al., 2017). Harrop et al. (2017) employed a manualised parent-coaching programme known as JASPER (Joint Attention, Symbolic Play, Engagement and Regulation; Kasari et al., 2021). JASPER is an empirically supported programme and teaches caregivers how to identify their child’s current play and social communication level (Harrop et al., 2017).

### Dependent Variables

The type of dependent variables (DVs) varied between studies in terms of target child outcomes. While all studies focused on targeting social communication behaviours, the behaviours targeted varied between each study. Targeted child outcomes included child verbal and non-verbal behaviours (such as gestures), behavioural requests for preferred items and activities, imitation skills (including object and gestural imitation), joint attention (including responding to bids for joint attention and duration of joint engagement), play skills and levels, and frequency of child expressive language.

All studies included a measure of parental fidelity on taught skills. Measures of fidelity varied between the studies and included both direct observation and observation via video by an independent rater. Including fidelity checks ensures that the data reported is valid and reliable and increases the face validity of the findings (St. Peter, 2023). All studies operationally defined the target behaviours parents were expected to engage in following coaching. This allowed for a measure of fidelity to be calculated, which was reported as a percentage in all studies. A fidelity of 80% was reported as the minimum standard parents had to achieve to be considered competent in implementing a targeted strategy in the majority of studies (Akemoglu & Tomeny, 2021; Erturk et al., 2021; Harrop et al., 2017; Zaghlawan & Ostrosky, 2016). In the Flippin (2019) study, the criterion was set at three consecutive sessions above the highest baseline level.

### Study Outcomes

Findings across all studies indicated that parent coaching was effective in teaching parents how to implement strategies to support their child’s social communication skills. All studies demonstrated positive effects for all parent-child dyads. As most studies employed single-subject research designs, effect sizes are unknown. Only one study employed a non-overlap measure (Erturk et al., 2021), which reported moderate to large effect sizes for both parent and child outcomes. In the large-scale group comparison study (Harrop et al., 2017), effect sizes were reported to be large and statistically significant. In addition, as the JASPER intervention group was compared to a parent psychoeducation group, it could be inferred that parent coaching is a more effective approach in providing parents with the skills needed to successfully implement strategies to support their child’s development. This was also evident in the studies which employed a parent training psycho-education phase followed by the parent coaching phase (Akemoglu & Tomeny, 2021; Erturk et al., 2021). The Flippin (2019) study also employed a measure of parental stress pre and post-intervention. The participating parents reported reduced stress related to some domains, including competence, while increased stress in the child domains of hyperactivity and distractibility were noted post-intervention.

### Social Validity

Three studies employed a social validity measure post-intervention (Akemoglu & Tomeny, 2021; Flippin, 2019; Zaghlawan & Ostrosky, 2016). One study utilised a questionnaire (Flippin, 2019), where the participating father rated the effectiveness of the coaching intervention and its components using a Likert scale. Overall, the father was satisfied with the intervention. Akemoglu and Tomeny (2021) conducted interviews with parents pre- and post-intervention. Before the intervention, the parents in the study identified that they would like their children to be able to communicate their wants and needs. They also highlighted that they valued collaboration in deciding on agreed-upon goals. Post-intervention, parents reported they were highly satisfied with the intervention, and that the procedures were easy to implement. They also reported satisfaction with their child’s communication outcomes. Similar findings were reported by parents in the Zaghlawan and Ostrosky (2016) study. Parents stated they became more aware of how to respond to their child’s needs and the need to allow for extra processing time.

## Discussion

The current review sought to synthesise the current research on parent coaching interventions for young autistic children. Specifically, the extent to which fathers are included in parent coaching interventions and the outcomes of such interventions were explored.

### Inclusion of Fathers

In line with the findings of the Flippin (2011) review, the current review underscored suspected gaps in the literature regarding caregiver gender. Only two father-child dyads were represented across all five studies, which featured a total of 94 parent-child dyads. The primary caregiver included in most studies was female, emphasising the ongoing lack of male caregivers in early intervention research. Indeed, this seems to be a part of a broader societal issue where fathers appear absent from numerous parenting interventions for children with developmental differences (Bagner, 2013). At a broader level, the research suggests that fathers have less involvement than mothers with disability and therapeutic support services, even when both are in full-time employment (MacDonald & Hastings, 2010; Roach et al., 1999). Traditionally, women have been regarded as the primary caregivers (Amato, 2018), leading to greater emphasis on their roles in research and service delivery for children with developmental differences. Despite evidence showing that the inclusion of both parents yields positive outcomes for child development, well-being, and family functioning, the current review noted that well-designed interventions incorporating fathers remain limited (Flippin & Crais, 2011; Tully et al., 2017; Wise et al., 2008).

Flippin (2019) was the only study that included a father as the sole participant and was weighted as “high” in terms of the relevance of the study in answering the research question (WoE C). The study was designed in line with paternal interaction styles. Zaghlawan and Ostrosky (2016) included one mother-child dyad and one father-child dyad in their study, and it was rated as “medium” in terms of WoE C. In contrast to Flippin (2019), the researchers did not adapt the intervention in line with paternal characteristics. In a systematic review conducted by Flippin and Crais (2011), suggestions were made on how to improve the accessibility of interventions for fathers. One of the main suggestions included incorporating play activities that reflect fathers’ play styles. According to the authors, fathers have different communication and play styles compared to mothers, and interventions involving fathers should consider this. For example, prior research has demonstrated that fathers tend to use more direct and complex language interactions with their children (Pancsofar & Vernon- Feagans, 2006, 2010). They also tend to engage in more physical play, such as chasing, tickling, and tossing their children in the air. These unique interactions have been shown to yield positive outcomes across key developmental areas, including emotional regulation and cognitive and language development (Flippin, 2019; Robinson et al., 2021). More specifically, the research indicates that movement-based or physical play has numerous benefits for autistic children, including enhanced motor skills, social interaction, communication, cognitive function, and self-regulation (Elbeltagi et al., 2023). Physical play fosters sensory integration between the central nervous system and the body, contributing to many of these positive outcomes (Elbeltagi et al., 2023; Pfeiffer et al., 2011). Thus, the physical play that fathers frequently engage in can provide opportunities for their children to experience diverse sensory input, such as touch, movement, and proprioception, which can aid in sensory processing. Additionally, some research has indicated that paternal interaction styles may be particularly effective in reducing externalising behaviours (Ting & Weiss, 2017). Therefore, considering father interaction styles in interventions may prove especially beneficial for autistic children, given the fundamental differences in social communication and emotional regulation abilities (American Psychiatric Association, 2022; Cibralic et al., 2019; Hansen et al., 2018; Hirschler-Guttenberg et al., 2015).

Studies that did not include fathers as participants offered no insight into how caregivers were chosen in the recruitment process. While recruiting families for such research can be challenging, efforts to balance gender among both child and parent participants should be implemented (Rich-Edwards et al., 2018). To tackle these issues, more diverse sampling strategies that specifically target fathers must be employed. Researchers may need to employ purposeful or convenience sampling to ensure fathers are adequately represented in parent samples. In addition, ensuring proper representation from various socioeconomic and cultural backgrounds is essential to explore whether parent coaching can be effective for fathers from differing backgrounds. Additionally, accommodating fathers’ work schedules has been noted as a vital factor in supporting fathers’ engagement with disability services (Curtiss et al., 2021). Work schedules should also be considered when delivering interventions to guarantee participation and minimise attrition.

Issues surrounding acceptability and feasibility for fathers should continue to be explored in an attempt to resolve their current under-representation in the field of autism research (Braunstein et al., 2013). Previous research has highlighted the need to work around some of the barriers that currently exist for fathers engaging with disability services. Curtiss et al. (2021) suggest that fathers may need to be offered more flexible appointments to suit their work schedules. In addition to adapting intervention goals to father interaction styles, Flippin and Crais (2011) also recommended making the context and activities for sharing information more father-friendly. When designing interventions, efforts should be made to ensure the materials and teaching style are accessible to the fathers. Previous research has also highlighted that services should emphasise that both parents are equally important to promote father engagement (e.g., Curtiss et al., 2021). Actively seeking to offer support to both mothers and fathers may be a step towards greater father engagement with disability services and align with a family-centered approach (Dunst & Espe-Sherwindt, 2016).

Another important point to note was that all the participants in the included studies were either mother-child dyads or father-child dyads. None of the included studies explored parent coaching with mother-father-child triads. According to McHale and Fivaz-Depeursinge (1999), from a family systems perspective, mother-father-child triadic interactions constitute many subsystems of the family, including the mother-child, the father-child and mother-father relationships. As such, mother-father-child triads have the potential to provide more diverse learning environments than each dyad in isolation (Lindsey & Caldera, 2006; Olhaberry et al., 2019). According to the literature, triadic relationships are important in a child’s social-emotional development (Feldman & Masalha, 2010). Through such interactions, a child learns how to navigate reciprocal interactions involving more than two people and many foundational social skills such as sharing and shifting attention, turn-taking, and expressing emotions (Franco et al., 2000; Venturelli et al., 2016). For typically developing children, the capacity to share attention with both parents simultaneously emerges from as early as 3 months of age (Siller, 2023). However, autistic children can take longer to acquire this skill, which is significantly associated with later social and communication development (Wong & Kasari, 2012). Therefore, including mother-father-child triads in parent coaching interventions may be particularly impactful for autistic children and promote a better understanding of reciprocal interactions and joint attention from an early age. Furthermore, as previously highlighted, mothers and fathers have unique and significant roles in their child’s development. Differences in interaction styles have been documented in the literature, particularly in the areas of play and language (Flippin, 2019; Flippin & Crais, 2011). The inclusion of mother-father-child triads in research may complement both parents’ interaction styles and provide a more enriching learning environment for the child.

Previous research has also indicated that interventions involving parent-child triads may benefit children experiencing difficulties in their social-emotional development (Olhaberry et al., 2019). However, few studies have explored the effects of parent coaching or training with the mother-father-child triad for autistic children. According to Siller, (2023), Oppenheim et al. (2023) have provided significant insights into the triadic relationship between autistic children and their parents in their recent study. They found that families in which both parents were insightful or responsive to their autistic child during play exhibited higher coordinated parental support than families in which neither or only one parent was insightful or responsive. This is significant as it underscores the necessity of including both parents in parent-training interventions to enhance coordination between parents in daily interactions. In previous research, Oppenheim et al. (2012) demonstrated that parental insightfulness lays the groundwork for sensitive or responsive parenting behaviours observed during interactions with their child (Siller, 2023). Working with the parent-child triad can promote better co-parenting, and Siller, (2023) has emphasised the need to further investigate the impact of the co-parenting alliance on the social-emotional development of autistic children.

### Intervention Outcomes

The second research question of this review was concerned with exploring the outcomes of parent coaching interventions which include fathers. This included overall effectiveness and the impact on parental stress. Overall, the findings from this systematic literature review supported the use of parent-coaching interventions for families of young autistic children, which is consistent with previous research (Deb et al., 2020; Sone et al., 2021; Ward et al., 2020). Positive outcomes for parents and children were reported across all studies. Of interest to this review, parent coaching was found to be effective in supporting parents to implement target strategies. Only two father-child dyads were represented in this review, which limits the generalisability of the findings. In the studies which did include fathers (Flippin, 2019; Zaghlawan & Ostrosky, 2016), parent coaching was found to be an effective approach. Flippin (2019) recruited a father as the sole participant and matched the target strategies to paternal interaction styles. Results demonstrated that the participating father learned all target strategies via coaching. While results demonstrated that fathers were successfully able to learn targeted skills, there was no comparison group or additional dyad that did not match target goals to father-interaction styles. Therefore, it is unknown if it is necessary to adapt intervention targets to this degree. One father-child dyad and one mother-child dyad were included in the Zaghlawan and Ostrosky (2016) study. In contrast to Flippin (2019), the researchers did not adapt the intervention in line with paternal characteristics. Despite the lack of accommodations for the participating father, similar findings were reported in terms of parent and child outcomes across both sets of parent-child dyads. While this limited amount of research suggests that parent coaching may be an effective teaching approach to use with fathers, more research is warranted to demonstrate its effectiveness and to ascertain if coaching needs to be adapted according to parental gender.

A secondary aim of this review was to explore the outcomes of parent coaching interventions, which included fathers on parental stress levels. Previous research suggests that the inclusion of fathers has the potential to buffer some of the stress experienced by mothers and improve overall family functioning (Braunstein et al., 2013; Laxman et al., 2015). Of interest to this review, one of the primary causes of stress reported by parents has been the management of their child’s intervention program (Iadarola et al., 2018; Parker et al., 2020). Only one (Flippin, 2019) of the two studies which included fathers as participants employed a measure of parental stress levels. Flippin (2019) included a measure of parental stress as rated by both parents pre and post-intervention. The participating parents reported reduced stress related to some domains, including competence, while increases in stress in the child domains of hyperactivity and distractibility were noted. The authors argued that the participating father may have become more aware of these characteristics while engaging more with his child during the intervention. However, it must be noted that differences in scores pre-post-intervention were only reported, and no statistical analysis was run. Therefore, it is not known if such differences in parental stress were statistically significant. Nevertheless, such findings may indicate the need to monitor parental stress levels throughout interventions and offer or signpost support where needed. Similar findings were also noted in a parent-mediated intervention conducted by Kasari et al. (2015), who suggested that taking on the role of therapist may have led to increased stress for some parents.

### Strengths and Limitations

This systematic review synthesised current research on parent-coaching interventions, looking specifically at the inclusion of fathers within such interventions. Findings highlighted that the experiences of fathers remain sparse in the literature despite whole-family approaches considered best practice (Flippin & Crais, 2011). It also highlighted the benefits of tailoring intervention targets to match fathers’ interaction styles, although the need for this remains unclear. In addition, the current review adds to the evidence base for parent coaching as an effective instructional approach that clinicians can utilise with both male and female parents. All articles were reviewed critically using Gough’s (2007) Weight of Evidence (WoE) framework. This entailed a rigorous review of each paper, allowing the researcher to determine which studies were the most relevant in answering the research questions. It also highlighted the need for more rigorous studies to be undertaken, as all studies were ranked as “medium” overall.

Despite the strengths of this review, a number of limitations should be taken into account when interpreting the findings. Firstly, only 5 studies were included in the final review. All of these studies were rated as “medium” in terms of their overall methodological relevance (WoE, D). Findings should, therefore be interpreted with caution. In addition, some methodological limitations were noted across studies. Of mention, not all included studies which employed a MBD collected data for 3 or more participants. Treatment effects observed in studies (Erturk et al., 2021; Flippin, 2019; Zaghlawan & Ostrosky, 2016) with less than 3 parent-child dyads may not generalise to similar populations and should be interpreted with caution (Cooper et al., 2014). Some of the single-subject designs also included a parent-training psychoeducational component before the implementation of parent coaching (Akemoglu & Tomeny, 2021; Erturk et al., 2021). Although the MBDs employed in these studies demonstrated a functional relationship between the introduction of the coaching intervention and improvements in parent and child outcomes, it is unclear which component of the intervention was responsible for behaviour change. As the phases were introduced sequentially, the true effect of the parent coaching phase in isolation is unknown. These studies, therefore scored “low” in terms of methodological relevance.

Overall, there was an inconsistency in the reporting of the intervention length. Therefore, it was difficult to determine what dosage, if any, may be the most effective. Studies which did not report the length of the intervention were rated lower in terms of methodological relevance. It was also difficult to determine treatment effect sizes to indicate the meaningful impact of studies. Employing the percentage of non-overlapping pairs (PND; Scruggs & Mastropieri, 2013) as a metric in single-subject studies would have allowed for treatment effects to be quantified and compared between studies. Calculation of this metric has been recommended as one step in the analysis of single-subject data, with minimal overlap between the phases indicating a strong effect (Scruggs & Mastropieri, 2013).

Lastly, it must also be noted that all of the included studies were conducted in the United States of America (USA) and, therefore, may not be representative of the entire population or be culturally sensitive. Cultural diversity within countries has become a focus of research, highlighting the need to be culturally sensitive to increasingly diverse populations (Lansford, 2022). Parenting practices are largely influenced by societal, religious and cultural norms (Ria Novianti & Nur Islami, 2023). While there has been a shift in the father’s role in Western society, this may not be reflective of non-Western societies. In most Eastern families, the father’s role has primarily been that of the breadwinner and an authoritarian figure (Ho et al., 2013). While there has been a shift in many countries, the traditional father role remains ingrained in many Eastern countries (Ria Novianti & Nur Islami, 2023). Therefore, consideration should be given to differences in parenting practices and preferences across and within cultures when reflecting on father participation in interventions and research. The concept of the ‘good father’ (Adler & Lenz, 2015) who is involved sensitively and accompanies their child in various activities may not be appropriate for all families depending on many factors, including cultural beliefs, family dynamics and socioeconomic status (Ho et al., 2013; Lansford, 2022; Planalp & Braungart-Rieker, 2016; Ria Novianti & Nur Islami, 2023). Such factors may explain the lack of father representation across cultures and differing backgrounds, while also highlighting the need to remain sensitive to diverse family systems.

### Conclusion and Implications for Practice

A thorough systematic review of the literature was conducted following PRISMA (2020) guidelines. Studies were critiqued both conceptually and methodologically using Gough’s (2007) Weight of Evidence (WoE) framework. This identified which studies were the most relevant in answering the target research questions. Key characteristics of the included studies were summarised in terms of participants, study design, dependent variables, intervention length, intervention outcomes and social validity measures.

Overall, coaching was reported to be an effective method to actively teach parents how to implement strategies in the context of the home environment. In comparison to other instructional approaches, such as didactic instruction, coaching has been found to have better outcomes for parents (Sone et al., 2021). Therefore, adopting a coaching framework may lead to the most favourable outcomes in parent-mediated interventions in the field of early intervention. Similar to findings from Pellecchia et al. (2020), common components of parent-coaching interventions were highlighted, including authentic learning opportunities, collaborative goal-setting, modelling or demonstration of the target skill, parent practice and feedback.

Lastly, central to this review, was the degree to which fathers were included in parent-coaching interventions. Only 1 study actively recruited a father as the sole participant (Flippin, 2019), while Zaghlawan and Ostrosky (2016) included one mother and one father dyad. The remaining 91 participants in the included studies were female. In terms of implications for future research and practice, recommendations for making future studies more amenable to fathers were suggested. This included adapting intervention goals in line with paternal interaction styles, adapting to father learning styles, offering flexible appointments and actively offering support to both mothers and fathers. In addition, studies would benefit from more diverse sampling strategies such as convenience or purposeful sampling to ensure fathers from varying backgrounds are adequately represented across parent samples.

While parent coaching yielded positive outcomes across studies, the limited number of studies that included fathers significantly restricts the robust and valid conclusions that can be drawn about the effectiveness of parent coaching for fathers. Only two fathers were represented in the included studies, creating a bias in the overall sample representation. The findings of this review may primarily reflect the experiences and outcomes of mothers as the primary caregivers rather than those of fathers. Consequently, the review’s findings may not be fully generalisable to fathers as a distinct group within the context of parent-coaching interventions. Given the small sample size, any reported effects may not be representative or robust and could be an artefact of sampling error. Future parent-coaching interventions targeted directly at fathers are needed to provide a more in-depth understanding of their unique experiences and to determine the true effectiveness of coaching for this population. Additionally, including qualitative data may offer a broader understanding of fathers’ experiences and support the identification of barriers and facilitators to their inclusion in research.

The review also highlighted another major gap within the area of parent-coaching interventions. Only mother-child or father-child dyads were represented in the included studies. None of the included studies explored parent-coaching with mother-father-child triads and its effects on child and parent outcomes. The benefits of interventions targeting child social-emotional skills with triads are documented in the literature (Olhaberry et al., 2019), and may be particularly beneficial for autistic children due to related differences in social-emotional development (Shire & Chang, 2022). It would also be interesting to explore the impact that including a triad would have on overall parental stress levels. However, overall, future research is warranted in this area.
